# Unraveling epigenetically deregulated lncRNAs FAM83A-AS2 and AC012213.1 as high-risk prognostic markers in lung adenocarcinoma

**DOI:** 10.1093/bib/bbaf631.069

**Published:** 2025-12-12

**Authors:** Syed Muktadir Al Sium, Mahafujul Islam Quadery Tonmoy, Sanjana Fatema Chowdhury, Fatema Tuz Zohra, Jean-Christophe Nebel, Farzana Rahman

**Affiliations:** Bangladesh Council of Scientific and Industrial Research, Dhaka 1205, Bangladesh; Bangladesh Council of Scientific and Industrial Research, Dhaka 1205, Bangladesh; Bangladesh Council of Scientific and Industrial Research, Dhaka 1205, Bangladesh; Bangladesh Council of Scientific and Industrial Research, Dhaka 1205, Bangladesh; Kingston University London, United Kingdom; Kingston University London, United Kingdom

## Abstract

**Background:**

Long non-coding RNAs (lncRNAs) are crucial regulators in cancer, yet their epigenetic control in Lung Adenocarcinoma (LUAD) remains underexplored.

**Aim:**

This study aimed to integrate multi-omics data to identify novel prognostic biomarkers and elucidate their potential mechanism.

**Methods:**

We analyzed RNA-Seq and Illumina 450 k methylation data from 473 LUAD and 32 normal The Cancer Genome Atlas (TCGA) samples. We performed differential expression and methylation analysis to identify candidate lncRNAs whose expression was negatively correlated with promoter methylation. The prognostic value was evaluated using Kaplan–Meier curves and multivariate Cox regression. A lncRNA-miRNA-mRNA regulatory network was constructed to investigate potential mechanisms. For validation, FAM83A-AS2 expression was quantified in a preliminary set of clinical blood samples using RT-qPCR.

**Results:**

Our multi-omics analysis identified two lncRNAs, FAM83A-AS2 and AC012213.1, whose high expression was driven by significant promoter hypomethylation and correlated with significantly lower patient survival. Differential analysis and correlation revealed promoter hypomethylation of specific CpG sites (cg19924352 for FAM83A-AS2; cg16648062 and cg20129213 for AC012213.1) drives their upregulation. Multivariate Cox regression confirmed their status as independent prognostic markers after adjusting for clinical covariates (HR = 1.55, p < 0.01 for FAM83A-AS2; HR = 1.30, p < 0.05 for AC012213.1), with strong diagnostic potential (AUC = 0.72). A regulatory network analysis implicated these lncRNAs in modulating key LUAD-associated genes like *RALGPS2*, *HOXA13* via miRNA MIR126 and MIR34C. Gene set enrichment analysis further linked these lncRNAs to fundamental molecular processes like chromatin modification and DNA methylation. Importantly, the pilot wet-lab validation on an initial set of clinical blood samples supported these findings, demonstrating a marked upregulation of FAM83A-AS2 in patients compared to healthy controls.

**Conclusion:**

This study presents FAM83A-AS2 and AC012213.1 as promising biomarkers for risk stratification and potential therapeutic targets in LUAD.

